# Trans-Spinal Direct Current Stimulation Targets Ca^2+^ Channels to Induce Persistent Motor Unit Responses

**DOI:** 10.3389/fnins.2022.856948

**Published:** 2022-04-25

**Authors:** Weiguo Song, John H. Martin

**Affiliations:** ^1^Department of Molecular, Cellular, and Biomedical Sciences, Center for Discovery and Innovation, City University of New York School of Medicine, New York, NY, United States; ^2^Institute of Bioelectronic Medicine, Feinstein Institutes for Medical Research, Manhasset, NY, United States; ^3^Neuroscience Program, Graduate Center of the City University of New York, New York, NY, United States

**Keywords:** motoneuron, trans-spinal direct current stimulation, persistent inward current (PIC), nimodipine, spinal cord, rat

## Abstract

Trans-spinal direct current stimulation (tsDCS) is a neuromodulatory approach to augment spinal cord activity to improve function after neurological disease and injury. Little is known about the mechanisms underlying tsDCS actions on the motor system. The purpose of this study is to determine the role for a persistent inward current (PIC)-like response in motoneurons in mediating tsDCS actions. We recorded single motor units from the extensor and flexor carpi radialis muscles in healthy sedated rats and measured unit activity changes produced by cervical enlargement cathodal and anodal tsDCS (c-tsDCS; a-tsDCS). Both c-tsDCS and a-tsDCS immediately increased spontaneous motor unit firing during stimulation. After c-tsDCS was stopped, spontaneous firing persisted for a substantial period (165 ± 5s), yet after a-tsDCS activity shortly returned to baseline (27 ± 7s). Administration of the L-type calcium channel blocker Nimodipine reduced spontaneous motor unit firing during c-tsDCS and blocked the persistent response. By contrast, Nimodipine did not change unit firing during a-tsDCS but the short persistent response was blocked. Computer simulation using a two-compartment neuronal model replicated the main experimental observations: larger and more persistent responses during and after c-tsDCS than a-tsDCS. Using reduced Ca^2+^ conductance to model Nimodipine action, a reduced response during c-tsDCS and elimination of the persistent response was observed. Our experimental findings, supported by computer simulation, show that c-tsDCS can target Ca^2+^ conductances to augment motoneuron activity. As tsDCS is well-tolerated in humans, this knowledge informs therapeutic treatment strategies to achieve rehabilitation goals after injury; in particular, to increase muscle force.

## Introduction

Spinal neuromodulation is a promising strategy to augment spinal cord activity to promote motor function after injury (for review, see Ievins and Moritz, 2017 and Jack et al., 2020). Several approaches have been implemented in animal models and humans. Non-invasive trans-spinal cord direct current stimulation (tsDCS) has the potential for promoting spinal motor function through its modulatory actions on sensory processing (Aguilar et al., 2011), reflexes (Winkler et al., 2010; Song et al., 2015; Mekhael et al., 2019), the motor cortex motor map (Ahmed, 2013a; Song et al., 2015), and enhancing spinal motor circuit function and motor output (Ahmed, 2011; Jankowska, 2017; Song and Martin, 2017). tsDCS has also been used for treating different neurological diseases and injuries (e.g., Picelli et al., 2015 and Lamy et al., 2021) and for pain control (Guidetti et al., 2021). The effects of tsDCS tend to be polarity dependent. Most studies showed cathodal (c)-tsDCS augments muscle activity driven synaptically by CNS stimulation and anodal (a)-tsDCS, either has no effect or suppresses activity (Bolzoni et al., 2013; Baczyk et al., 2014; Bolzoni and Jankowska, 2015; Knikou et al., 2015; Song et al., 2016). However, the effects are not entirely consistent with reports of anodal facilitation (Ahmed, 2016; Baczyk et al., 2019, 2020b). Many questions remain unanswered about the mechanisms underlying tsDCS neuromodulation of spinal circuits, including the neuronal targets engaged by tsDCS (synaptic vs. intrinsic; interneurons and motoneurons) and the molecular underpinnings. Modeling and experimental studies suggesting that spinal nerve roots and spinal neurons—in particular, motoneurons (Elbasiouny and Mushahwar, 2007)—play a role in augmenting motor output with polarizing spinal neuromodulation (Hernandez-Labrado et al., 2011; Jankowska, 2017). Here we focus on the spinal motoneuron, through single motor unit recording, as a potential target for tsDCS.

Spinal motoneurons are located ventrally in the gray matter, with large cell bodies and extensive dendritic arbors, especially long dorsal branches (Stifani, 2014). Motoneurons have a myriad of synaptic and intrinsic molecular mechanisms enabling flexible excitability regulation (Heckman et al., 2003; Stifani, 2014). Voltage-dependent persistent inward currents (PICs or plateau potential) are leveraged by the motor systems to modulate motoneuron activity (Heckmann et al., 2005) and, as such, may be a potential target for neuromodulatory regulation of muscle functions. PICs are long-lasting responses that are primarily mediated by an L-type Ca^2+^ conductance (Hultborn, 2002). PICs increase motoneuronal firing rate and prolong the firing duration after stimulation has ended. PICs play important motor control roles during normal motor behavior (Kiehn and Eken, 1989; Gorassini et al., 1999; Gorassini et al., 2002a,b). After spinal injury, PICs become dysregulated and contribute to hyperreflexia and spasms (Heckman et al., 2003; Murray et al., 2010; Marcantoni et al., 2020). C-tsDCS enhances motor cortex-evoked muscle responses (MEPs) during (Ahmed, 2011; Knikou et al., 2015; Song et al., 2015) and after stimulation (Ahmed, 2011; Song et al., 2016). For transcranial DC stimulation, the capacity for prolonged MEP enhancement is thought to reflect activity-dependent plasticity (e.g., LTP) (Fritsch et al., 2010; Monte-Silva et al., 2013; Lefaucheur et al., 2017). Whereas LTP-like mechanisms may be engaged under some conditions, we propose that PICs—an intrinsic membrane mechanism—are a target of tsDCS neuromodulation. We focus on PICs because modeling of the neuronal response to DC stimulation indicates that the neuronal somato-dendritic membrane polarizes within the applied electric field (Bikson et al., 2004); especially the long dendritic processes of motoneurons (Elbasiouny and Mushahwar, 2007), which is where L-type Ca^2^+ channels are predominantly localized (Heckmann et al., 2005).

In this study, we examine changes in spontaneous wrist muscle single motor unit firing induced by tsDCS as a means to understand its actions on motoneurons (Gorassini et al., 1999). We hypothesize that an increase in spontaneous motoneuron activity, assessed non-invasively using single motor unit recording, will occur during and persist following c-tsDCS. Persistent motor unit firing after stimulation is consistent with an increase in PICs in motoneurons (Gorassini et al., 1999). We recorded single unit responses in the extensor/flexor carpi radialis (ECR/FCR) muscles in sedated rats before, during, and following c-tsDCS and a-tsDCS of the cervical spinal cord (Song et al., 2015). We show that both c- and a-tsDCS consistently increased motor unit firing during stimulation but only c-tsDCS produced a robust persistent response. Pharmacological blockade of L-type calcium channels using Nimodipine, an FDA-approved L-type Ca^2+^ antagonist that has been used to mitigate spasticity in an animal injury model (Marcantoni et al., 2020), eliminated the persistent response. We also provide support for the major experimental observations using a computer simulation based on a two-compartment neuron model (Booth et al., 1997; Kurian et al., 2011; Kim, 2017; Lafon et al., 2017). Our study suggests that tsDCS modulates spinal activity, in part, by differentially acting on the dendrite and soma of motoneurons, with c-tsDCS preferentially activating Ca^2+^ channels in the dendrite to produce PICs, while a-tsDCS preferentially depolarizes the soma.

## Materials and Methods

The effects of c- and a-tsDCS on single motor unit firing and Nimodipine channel blocking experiments were examined in sedated rats (Sprague-Dawley; *n* = 5; 280–320 g). Experimental design incorporated repeated testing of animals with both c-tsDCS and a-tsDCS (two sessions). We allowed for a period of at least 7 days between each experiment in the same animal for any carryover effects to dissipate. All experiments were approved by the IACUC of the City University of New York Advanced Science Research Center.

### Animal Preparation

Rats were sedated with ketamine (80 mg/kg, IP) during stimulation and recording for all experiments. Animals were placed on a table in a prone position with normal body temperature maintained with a heating blanket. The forepaw was placed in a posture that extended the wrist without inducing background electromyographic (EMG) activity. The sedation level was checked by monitoring the breathing rate, spontaneous vibrissae whisking, and hindlimb withdrawal to toe pinch. Supplemental doses of ketamine (25 mg/kg) were administered as needed to maintain the required sedation during the experiment. After final testing, rats were euthanized by IP administration of a Ketamine/Xylazine overdose.

### Trans-Spinal Direct Current Stimulation

**FIGURE 1:**
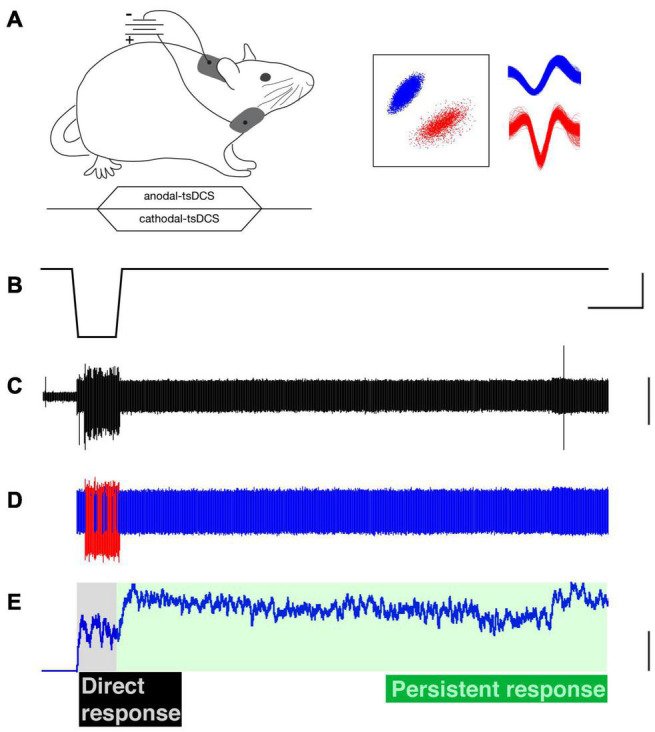
Trans-spinal direct current stimulation (tsDCS) and single motor unit recording. **(A)** Schematic of experimental setup (top) and time-course of tsDCS (bottom). tsDCS was delivered through patch electrodes with the active electrode placed dorsally over the C4 to T1 vertebrae and the return electrode (i.e., opposite polarity) placed over chest. tsDCS was ramped over a 3s period to the maximal current, which was maintained for 20s, and ramped back to zero during a 3s period. **(B)** Cathodal-tsDCS waveform. **(C)** Raw electromyographic (EMG) recording (ECR muscle). **(D)** Raw EMG activity was sorted into single motor units using a PCA-based identification. Representative examples of two single motor units (red and blue). Their corresponding waveforms are shown in part **(A)** (right). **(E)** The firing rate was used to characterize the firing pattern; only the firing rate of the blue-colored unit is shown. The direct response is during the stimulation period (gray shading), whereas the persistent response is after tsDCS is turned off (green shading). Calibrations. **(B)** 25s, 2.5 vDC. C. 0.2 arbitrary units. **(E)** 25 Hz.

Two 1.5 cm × 2 cm gel patch electrodes (StimTent Com.) were used to deliver tsDCS. First, the hair over the dorsal neck and chest of the animal was shaved and further removed with Nair. Second, electrically conductive adhesive was sprayed over the contact surface of electrodes, to optimize and stabilize electrical conduction between the skin and electrodes, and then applied to fixed locations in each animal (dorsally, over C4-T1 and the other was placed over the chest; Figure 1A) according to the results of prior modeling experiments (Song et al., 2015). The polarity of tsDCS stimulation was referenced to the dorsal electrode (cathode). tsDCS was generated with an analog isolated stimulator (model 2200, A-M Systems), that was controlled by an analog output channel of an experimental control and data acquisition system (CED, Inc., Cambridge, United Kingdom). Twenty seconds of c-tsDCS (-3 mA) or a-tsDCS (+3 mA) was tested with a 3s ramp for both up and down phases (Figures 1A,B). To avoid the effect of cathodal stimulation on the anodal response, and vice versa, we typically recorded unit activity either in response to one or the other polarity during a given day. The tsDCS intensity of ±3 mA was chosen based on our previous observation and a modeling study indicating consistent effects on MEP enhancement with c-tsDCS (Song et al., 2015). This intensity (current density: 1 mA/cm^2^; total charge density: 26 mC/cm^2^) could induce consistent effects, and is below the threshold value for tissue damage (Yuen et al., 1981; McCreery et al., 1990; Liebetanz et al., 2009). This current produced reddening of the skin, especially at the edges of the electrode, but no damage.

### Single Motor Unit Recording

Pairs of PFA-insulated stainless steel microwire electrodes (0.002” diameter, A-M Systems) were deinsulated at the tip (about 1 mm) and were inserted into the flexor carpi radialis (FCR) and/or extensor carpi radialis (ECR) muscles. EMGs from the recorded muscles were filtered (300–5000 Hz) and amplified (×1,000), and then digitized at a sampling frequency of 10 kHz with an acquisition system (CED, Inc). Raw EMG records (Figure 1C) were analyzed offline for single motor units. Spikes of single motor units were sorted according to principle component analysis (PCA) or template matching from the recorded EMG signals (Figures 1C,D) and further manually cluster-cut with a customized script written with Spike 2 (Figure 1A, inset). The firing rate of each sorted single motor unit was smoothed with a 1s sliding window, and the ensemble response was constructed from the smoothed firing of all the recorded motor units during c-tsDCS and a-tsDCS. The majority of the recorded motor units were from the ECR muscle, with additional recordings from FCR (c-tsDCS: ECR, 16; FCR, 4) and 22 (a-tsDCS ECR, 17; FCR, 5). No differences were found between ECR and FCR responses; thus, we combined data from the two muscles.

### Classification of Motor Unit Response Pattern

We classified two response components of motor units (Figure 1E). The (1) direct response corresponds to unit firing during the ramp and plateau phases of tsDCS and (2) the persistent response corresponded to firing after the tsDCS had returned to baseline. Motor units were characterized according to the following five metrics: (1) the percentage of units that showed a direct response during tsDCS; (2) the firing rate during tsDCS; (3) the percentage of units that showed persistent firing after tsDCS; (4) persistent firing duration, which is the duration of elevated firing (over baseline) after cessation of tsDCS; and (5) persistent response gain, which is the ratio of the area of the persistent unit response over area of the direct response.

### Ca^2+^ Channel Blockade

Since PICs are primarily mediated by an increase in inward calcium current (Li and Bennett, 2003), we used the L-type calcium channel blocker Nimodipine (125 mg, Sigma Inc., Burlington, MA, United States, USP grade) to determine if the persistent effect of tsDCS on motor unit firing is mediated by a calcium PIC. Nimodipine is a non-selective L-type Ca^2+^ channel blocker (Carlson et al., 2020). Nimodipine was dissolved in vehicle (ethanol, DMSO, polyethylene glycol and saline in the following proportions: 1:1:8:10), and then sterilized using a syringe filter (30 μm), and stored in a sterile dark bottle at room temperature. It was injected into the tail vein over a 3–5 min period (5 mg/kg). Induction time for the drug is approximately 30 min after IP injection (Marcantoni et al., 2020). We choose to use IV administration to obtain a faster response. We examined open field behavior after Nimodipine administration and did not observe any behavioral changes at this dose (data not shown).

### Two-Compartment Computational Model to Predict Effect of Trans-Spinal Direct Current Stimulation on Motor Unit Firing

Experimental observations were compared with a computer simulation using a two-compartment neuron model. With this model, PICs were previously found to be changed after spinal cord injury (Kurian et al., 2011). This model also has been used to study normal spinal motor neuron behavior (Booth et al., 1997; Kim, 2017). The motor neuron was modeled with two simplified compartments: dendritic and axosomatic. Both compartments are described by active and passive conductances following the Hodgkin-Huxley formalism. The somatic compartment contains ionic conductances that generate action potentials. The dendritic compartment contains conductances responsible for plateau potentials for generating PICs. Voltage-dependent Na and Ca^2+^ channels were modeled in the dendrite only. The model included the following dendritic conductances: Na_*p*_ (Sodium, persistent); K_*Ca*_ (Potassium, Ca^2+^ dependent); Ca_*p*_ (Calcium, persistent); L (maximal leakage conductance). The following somatic conductances were modeled: Na (maximal Sodium conductance); Ca_*N*_ (Calcium, N-like); L (maximal leakage conductance); K_*Ca*_ (Potassium, Ca^2+^-dependent); K_*dr*_ (Potassium, delayed rectified). All channel dynamics and transition rates, along with conductances for the model, were set according to published values (Kurian et al., 2011). tsDCS was modeled as an external electrical field, as previously described (Lafon et al., 2017). During direct current stimulation, the neuron will polarize along the direction of the electric field. This results in opposite changes in membrane potential at the dendritic and somatic sties. The numerical solutions of the model were computed using ode15s function of Matlab (Mathworks, Inc., Nattic, MA, United States).

### Statistical Analysis

The differences between two conditions (pre vs. post Nimodipine or a-tsDCS vs. c-tsDCS) within each group were assessed by parametric tests (*t*-test, MATLAB). We performed a linear regression. The non-parametric Kolmogorov–Smirnov test was used to assess differences in the distributions of two groups (K-S test, MATLAB). The significance level was set at 0.05. All data analyses were performed using MATLAB (The Math Works). In accordance with the Journal’s Data Availability Declaration, for quantitative presentation of data, all values are shown.

## Results

### Effects of Trans-Spinal Direct Current Stimulation on Motor Unit Activity

**FIGURE 2:**
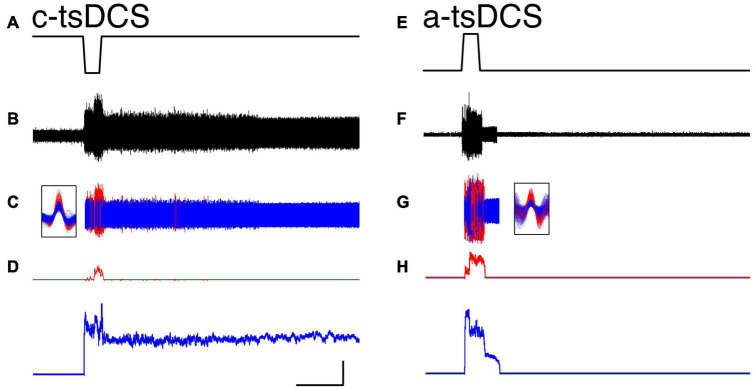
Representative single motor unit responses during trans-spinal direct current stimulation (tsDCS) [**(A,E)**; ± 3 mA] during cathodal-tsDCS (c-tsDCS) **(A–D)** and anodal-tsDCS (a-tsDCS) **(E–H)**. Raw EMG activity **(B)**, single motor unit activity [**(C)**; red, blue for each unit], and response histograms **(D)** are shown. The unit waveforms were sorted from the raw EMG recording. Both c-tsDCS and a-tsDCS induced single motor unit responses during the stimulation period (direct response). Whereas responses persisted after stimulation stops for both polarities, c-tsDCS **(B–D)** evoked long-duration persistent responses. During a-tsDCS **(E–H)**, there was a brief persistent response. Note, the responses were truncated at 380s; the activity of the blue unit in A persisted for a total of approximately 532s. Calibration: 50s, 50 Hz.

We first distinguished the effect of tsDCS during the period of stimulation (direct response) from unit firing that was changed after tsDCS ended (persistent response). A representative ECR EMG recording is shown (Figures 2A,B) and two units activated in response to c-tsDCS were isolated (Figure 2C). Motor unit activity is transformed into a continuous frequency plot (Figure 2D). C-tsDCS produces a robust increase in firing during stimulation for both units. Remarkably, the firing of one of the motor units (blue) is maintained for 210 seconds after stimulation ends. Whereas a-tsDCS (Figure 2E) also produced a direct effect, there was little or no maintenance of persistent unit firing (Figures 2F–H). Note, the delay in firing from stimulus onset of the red unit is due to the current ramp and the response threshold for the particular unit. In addition to showing modulatory effects on spontaneous single motor unit activity by tsDCS, we demonstrate that c-tsDCS produces a persistent response similar to the increase in motoneuronal firing observed during activation of a PIC. We also noticed for each of these units that c-tsDCS induced a stronger effect than a-tsDCS during testing (data not shown).

### Direct Response Induced by Cathodal and Anodal Trans-Spinal Direct Current Stimulation

**FIGURE 3:**
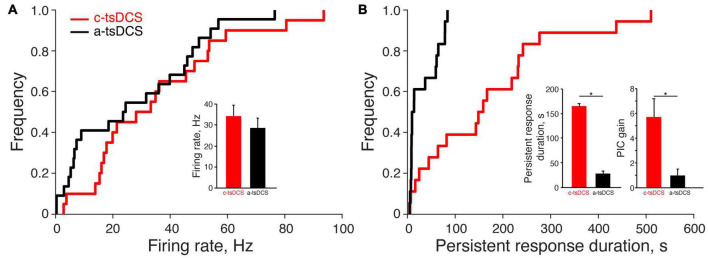
Effect of trans-spinal direct current stimulation (tsDCS) on the motor unit direct and persistent response. **(A)** The cumulative distribution functions of the direct response for anodal-tsDCS (a-tsDCS) and cathodal-tsDCS (c-tsDCS) were not significantly different (K-S test, *p* = 0.22). Inset plots mean firing rates during both c-tsDCS and a-tsDCS, which were not significantly different (*t*-test, *p* = 0.29). **(B)** The cumulative distribution function of persistent response duration was significantly different between c-tsDCS and a-tsDCS (K-S test, *p = 0.0005). The inset plots mean PIC duration, which was significantly longer for c-tsDCS (*t*-test, *p = 0.0002) and PIC gain, was significantly longer and stronger during c-tsDCS than during a-tsDCS (*t*-test, *p = 0.006).

We recorded from a total of 20 motor units (ECR, 16; FCR, 4) during c-tsDCS and 22 (ECR, 17; FCR, 5) during a-tsDCS in five rats across multiple sessions with at least 7 days between any two sessions in the same animal. No differences were found between ECR and FCR responses; thus, we combined date from the two muscles. The percentage of units that had a direct response was slightly higher in c-tsDCS (100%) than a-tsDCS (86%). The cumulative distribution function (Figure 3A) plots the mean firing rate during the stimulation period. There was no significant difference between c-tsDCS and a-tsDCS distributions (K&S test), nor was there a difference in mean firing rate (Figure 3A, inset; *t*-test, *p* > 0.05).

### Cathodal Trans-Spinal Direct Current Stimulation Persistent Response Is Greater Than the Anodal Persistent Response

Similar to the direct response, most units showed some persistent response with either polarity (90% in c-tsDCS vs. 77% in a-tsDCS). However, the cumulative distribution functions of persistent firing duration were significantly different for the two polarities (Figure 3B; K-S test, *p* < 0.05) and show a wide range of response durations. The mean duration of the persistent increase in motor unit firing was approximately six-times longer after c-tsDCS than a-tsDCS (Figure 3B, inset; *t*-test, *p* < 0.05). We computed a measure of the gain of the persistent response for each unit (Figure 3B; persistent response area divided by direct response area) and found this was also approximately 6 times greater for cathodal than anodal stimulation (*t*-test, *p* < 0.05). Our findings suggest that c-tsDCS activates a PIC to prolong the elevated motor unit activity beyond the stimulation period.

### Persistent Response Is Eliminated by Calcium Channel Blockade

**FIGURE 4:**
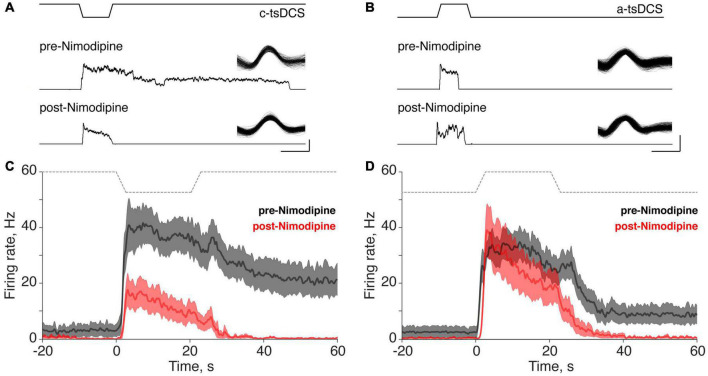
Effect of the L-type calcium channel blocker Nimodipine on direct and persistent responses of trans-spinal direct current stimulation (tsDCS) (±3 mA). **(A,B)** Typical responses of single motor units before and after Nimodipine application during cathodal-tsDCS (c-tsDCS) **(A)** and anodal-tsDCS (a-tsDCS) **(B)**. From top to bottom, we show the tsDCS waveform, and pre- and post-Nimodipine unit histograms. The inset shows the waveform of the isolated motor unit. C-tsDCS induced both direct responses and persistent responses. For c-tsDCS, Nimodipine reduced the direct response and blocked the persistent response. Nimodipine did not change the response during a-tsDCS stimulation but did block the brief persistent response. The single unit waveforms were identified from the sorted single motor units from the raw data and the smoothed firing rate (1s window) before and after Nimodipine. **(C,D)** The ensemble response from all the recorded units shows that Nimodipine completely blocked the persistent response and also suppressed the direct response during c-tsDCS **(C)**. By contrast, a-tsDCS did not significantly affect the direct response during a-tsDCS **(D)**. The thick lines and shaded area **(C,D)** represent mean ±SE, and gray lines indicate the applied tsDCS.

To better understand the underlying mechanism of the persistent increase in motor unit firing during tsDCS, and to distinguish the mechanisms underlying the direct from the persistent responses, we administered the L-type Ca^2+^ channel blocker Nimodipine (5 mg/kg, IV; tested 15 min post-injection). We determined if Ca^2+^ channel blockade changed the properties of the direct and persistent responses. For the two units shown, the direct responses to c-tsDCS and a-tsDCS were similar (Figures 4A,B), whereas the persistent response was only produced by c-tsDCS. Nimodipine completely blocked the persistent response in the motor unit, with a smaller effect on the direct response (Figure 4A). Although there was no persistent response produced by a-tsDCS in the unit shown (Figure 4B) there was a small reduction in firing during the direct response. Interestingly, there was a small increase in the direct response duration but insufficient to produce a persistent effect after stimulation ceased. Ensemble responses (Figures 4C,D; 20 motor units in c-tsDCS and 22 in a-tsDCS) show complete elimination of the persistent response after c-tsDCS with approximately a 50% decrease in the peak of the direct response after Nimodipine (Figure 4C). In contrast, Nimodipine had a minimal effect on the direct response to a-tsDCS and the small persistent response that was produced, was eliminated (Figure 4D).

**FIGURE 5:**
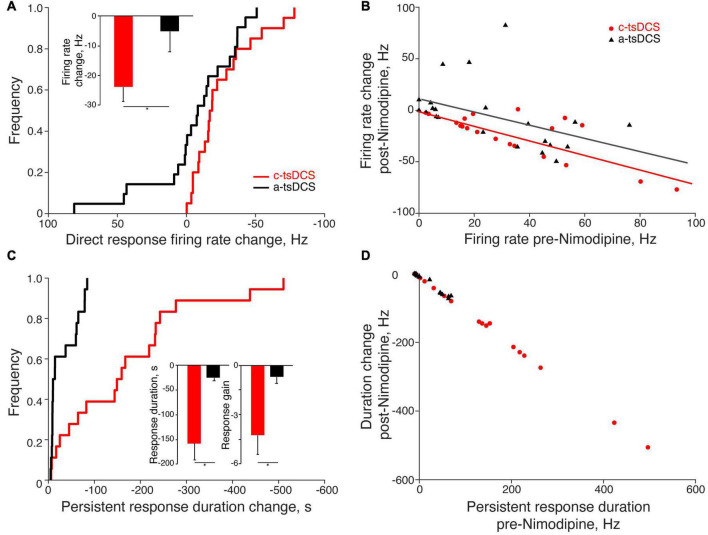
Quantification of the effects of Nimodipine on the direct and persistent responses of single motor units. **(A)** The change of the direct response firing rate (post-Nimodipine response minus pre-Nimodipine response) was significantly larger for cathodal-tsDCS (c-tsDCS) than for anodal-tsDCS (a-tsDCS) (K-S test, *p = 0.046) as shown in the cumulative distribution histogram. The inset plots the reduction in the firing rate during the direct response; the reduction was significantly greater for c-tsDCS than for a-tsDCS (*t*-test, *p = 0.03). **(B)** The blockade effect of nimodipine was correlated with the direct response amplitude; each data point represents a single motor unit (c-tsDCS regression line: slope: −0.70, R-sq: 0.59, *p* = 0.0001; a-tsDCS regression line: slope −0.62, R-sq:0.20, *p* = 0.04). **(C)** The change in duration of the persistent response (post-Nimodipine response minus pre-Nimodipine response) was significantly greater for c-tsDCS than for a-tsDCS (K-S test, *p = 0.0005). The insets plot the reduction in the duration (left), and gain (right) of the persistent response for both c- and a-tsDCS; the blockade effect of Nimodipine was significantly stronger for c-tsDCS than for a-tsDCS (*t*-test, duration *p = 0.0002; gain *p = 0.04). **(D)** The effect of blockade was correlated with the persistent response strength; each data point represents a single motor unit (c-tsDCS regression line: slope: −1.00, R-sq: 1.00, *p* = 1e-28; a-tsDCS regression line: slope −0.92, R-sq:0.98, *p* = 3e-18).

To better reveal how variable the response to Ca^2+^ channel blockade is, we plot the cumulative distribution function for the change in the direct response firing rate (Figure 5A; pre-Nimodipine minus post-Nimodipine). The plot shows a consistent reduction with c-tsDCS (rightward shift) and a mixture of symmetrical changes around zero for a-tsDCS. The effect of blockade on the two polarities was significant (K-S test, *p* < 0.05; Figure 5A). The percentage of motor units showing a direct response was significantly decreased after Nimodipine for c-tsDCS (−50%). than for a-tsDCS (−11%). The mean reduction in firing rate of the direct response after blockade was also significantly different between c-tsDCS and a-tsDCS (*t*-test, *p* < 0.05; Figure 5A, inset). The effect of calcium channel blockade on the direct response was linearly correlated with the mean firing rate of the direct response (Figure 5B). The larger the direct response the stronger the blockade effect for both c-tsDCS (slope: −0.70, R-sq: 0.59; *p* < 0.05) and a-tsDCS (slope: −0.62, R-sq 0.20; *p* < 0.05).

After Nimodipine administration, the duration of the persistent response showed a consistent reduction for the c-tsDCS and, not surprisingly, a negligible change for a-tsDCS (Figure 5C). Most of the persistent responses of motor units were blocked by Nimodipine for both c-tsDCS (−67%) and a-tsDCS (−58%). Similar to the direct response, the blockade effect was linearly correlated with the strength of persistent response: the larger the persistent response the stronger the blockade effect for both c-tsDCS and a-tsDCS (Figure 5D). The slope of this relationship is negative one (R:1, *p* < 0.05), thus affirming the strong L-type calcium channel dependence. Not surprisingly, the blockade effect was stronger for c-tsDCS than for a-tsDCS; for both persistent response duration and persistent response gain (Figure 5C, insets). Our findings reveal a robust effect of Nimodipine on abrogating the persistent response produced by c-tsDCS suggesting that the persistent response is mediated by a Ca^2+^ PIC.

### Two-Compartment Simulation

**FIGURE 6:**
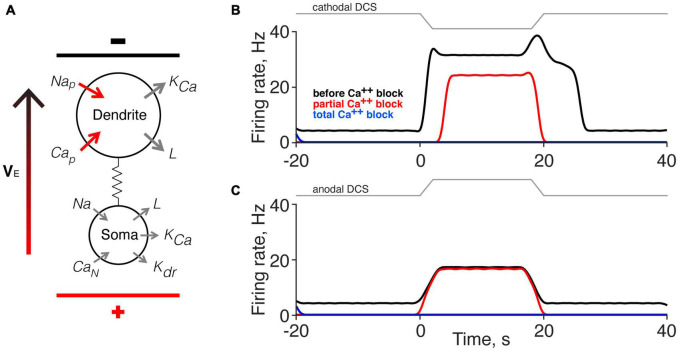
Computer simulation of the effects of direct current stimulation (DCS) on the direct and persistent responses. **(A)** Schematic of the motoneuron model (Booth et al., 1997). Applied DCS was modeled as an external electrical field (V_*E*_), which induces a current between soma and dendrite. The configuration in the model illustration corresponds to cathodal-tsDCS (c-tsDCS) in the experiment. Ca_*p*_, Calcium, persistent; Ca_*n*_, Calcium, N-like; K_*Ca*_, Potassium, Ca^2+^ dependent; K_*dr*_, Potassium, delayed rectified; L, maximal leakage conductance; Na, maximal Sodium conductance; Na_*p*_, Sodium, persistent. **(B,C)** Computer simulation for response change produced by c-tsDCS **(B)** and anodal-tsDCS (a-tsDCS) **(C)**. The responses during c-tsDCS were stronger than that during a-tsDCS. Only c-tsDCS induced a persistent firing of motor units. The responses during c-tsDCS showed calcium channel-dependent; there was a reduction in the direct response and elimination of the persistent response. In contrast, the responses during a-tsDCS were not calcium channel dependent. Total calcium channel blockade eliminated all responses.

We used a computer simulation of a two-compartment neuron model (Booth et al., 1997; Kurian et al., 2011; Kim, 2017) to inform our finding that the persistent motor unit response is mediated by a Ca^2+^ PIC. Figure 6A shows a schematic representation of the neuron model as well as its coupling to the external field through the extracellular potential difference *V*_*E*_. Voltage-dependent sodium channels and calcium channels were modeled in the dendrite only (Heckman et al., 2003; Murray et al., 2010; Marcantoni et al., 2020). The motor neuron time constant and conductance parameters used in our simulation were chosen from a published paper (Kurian et al., 2011; Mekhael et al., 2019). The strength of the external applied direct current stimulation (DCS) was modeled according to our published data (Song et al., 2015), and the effect of calcium channel blockade produced by Nimodipine was modeled by a partial Ca^2+^ conductance block. We used a 10% reduction (from 0.35 to 0.315); however, progressive Ca^2+^ conductance reduction did not systematically and linearly reduce the direct response.

The model replicated the key features of the experimental findings. Parameters set for the model account for the presence of spontaneous activity (black line before and after DCS). Modeling cathodal DCS produced both direct and persistent responses (Figure 6B), similar to our experimental findings using c-tsDCS (Figures 2D, 4C, pre-Nimodipine). Partial Ca^2+^ channel blockade reduced the direct response and eliminated the persistent response, which is similar to our experimental findings (Figures 2D, 4C). By contrast, modeling anodal DCS produced only a direct response and this was unaffected by partial Ca^2+^ channel blockade (Figure 6C). Additionally, the direct response modeled by anodal DCS is smaller than during cathodal DCS. Results of our simulation suggest that both c-tsDCS and a-tsDCS activate motor units during tsDCS and this direct response is a balance between activation of the dendrites and the soma, whereas the persistent response is only present during c-tsDCS and is modulated by dendritic calcium channels.

## Discussion

Although the mechanisms of action of different neuromodulation strategies on spinal circuits are yielding to experimental and computational approaches, we are far from having a sufficient understanding to inform therapeutic decisions. We focused on tsDCS, a non-invasive neuromodulatory tool with the potential for improving motor function after neurological disease and injury (Rahman et al., 2013; Ahmed, 2014; Song et al., 2015, 2016; Mekhael et al., 2019; Abualait, 2020; Lamy et al., 2021). We show a novel action on motoneurons using single motor unit recordings; c-tsDCS produces a robust augmentation of spontaneous single motor unit firing that persists after stimulation stops. This response is blocked with Nimodipine, showing that L-type Ca^2+^ channels contribute to this neuromodulatory action and that PICs in motoneurons participate in the persistent response. A two-compartment somato-dendritic neuron computer simulation supports these experimental results. Knowledge that the actions of c-tsDCS produces spinal PIC-like responses informs therapeutic strategies for using tsDCS to promote function after injury.

### Are Spinal Cord Neurons a Target Engaged by Trans-Spinal Direct Current Stimulation to Induce Persistent Motor Unit Firing?

It is not understood if the excitatory actions of c-tsDCS reflect membrane depolarization of spinal cord neurons or if it indirectly affects spinal neurons by depolarizing the axons of peripheral somatic sensory afferents and other intraspinal axons and terminals (Bolzoni and Jankowska, 2015; Formento et al., 2018), or supraspinal processing through ascending synaptic effects (Ahmed, 2013b; Bocci et al., 2014, 2015). Some studies suggest that a-tsDCS may affect axonal conduction, while c-tsDCS modulates interneuronal spinal networks (Bocci et al., 2014). Locally-applied (intraspinal) DC fields, in the microampere range, can enhance the excitability of intraspinal afferent fiber terminals (Bolzoni and Jankowska, 2015) and epidural direct current stimulation produces similar effects (Jankowska et al., 2017). Whereas there are multiple targets that could contribute to persistent firing, the well-known role for motoneuronal PICs is likely to be a dominant factor. We found that both cathodal and anodal stimulation produced a direct facilitatory effect (albeit a weaker anodal than cathodal response), but only cathodal stimulation produced a persistent response. Perhaps the absence of a direct cathodal facilitation with intracellular motoneuron recordings (Baczyk et al., 2019, 2020a) reflects the deeply anesthetized preparation. It is known that pentobarbital anesthesia does not support PICs (Button et al., 2006); there may be other molecular targets of tsDCS that are affected by deep anesthesia. The discrepancy may also be due to misalignment of the polarization field and dendrite orientation or sampling the activity of large motoneurons (Ahmed, 2016). Note that the enhanced motor unit firing during a-tsDCS is not significantly reduced by nimodipine, suggesting a source for activation other than the L-type Ca^2+^ channel. It is likely that there are multiple neural targets, especially if one considers both the direct and persistent effects. Further experiments and more sophisticated modeling [i.e., multi-compartment (Elbasiouny and Mushahwar, 2007)]; are needed to dissect the specificity of tsDCS actions. However, based on pharmacological blockade we propose that the L-type Ca^2+^ is a key mediator of the PIC.

### Molecular-Level Mechanism of Trans-Spinal Direct Current Stimulation on Motoneuron

The effects of tsDCS, in both human and animal models, has been studied predominantly at the level of changes in motor cortex-evoked motor output [e.g., MEPs; also spinal LFPs; Ahmed (2011); Knikou et al. (2015), Song et al. (2015), and Song and Martin (2017)]. Changes in motoneuron excitability may reflect membrane polarization in response to the external applied electrical field, but also can be modulated through different ion channels (Booth et al., 1997; Rahman et al., 2013). Thus, changes in spontaneous activity of single motor units can provide insight into the molecular mechanism of tsDCS on motoneurons.

Similar to the facilitatory effect on motor cortex MEPs (Song et al., 2015), we also find facilitation of the spontaneous single motor unit response during c-tsDCS. However, in contrast to prior findings that a-tsDCS reduced motor cortex-evoked MEPs, the direct response during a-tsDCS increased, albeit less than for cathodal stimulation. This suggests differential actions of a-tsDCS on synaptically-evoked MEPs (Bolzoni and Jankowska, 2015) and on spontaneous single motor unit activity during the direct response (Bolzoni and Jankowska, 2015). Here, we show that tsDCS modulation of spontaneous activity is driven by intrinsic excitability mechanisms; in particular, the L-type calcium channel.

Polarization of hippocampal neurons in brain slices affects both spontaneous firing rate and synaptic efficacy (Lafon et al., 2017). We also found that tsDCS could modulate motoneuron PIC gain, with the c-tsDCS effect being substantially greater than that of a-tsDCS. The two-compartment model showed that the PIC arises predominantly from dendritic Ca^2+^ influx (Kurian et al., 2011). Although c-tsDCS could hyperpolarize the somatic membrane potential (i.e., DC-induced neuronal polarization), the strong inward current from the dendrite would be expected to depolarize the soma, causing a plateau potential. Our findings point to the principal mechanism for this augmentation as activation of the voltage-dependent calcium channel within the dendritic compartment to produce a PIC-like motoneuronal response.

### Limitations of the Two-Compartment Model

We hoped to model the question of whether tsDCS field polarization, through actions on dendritic Ca^2+^ channels and PICs, can lead to persistent motor unit firing. Whereas tsDCS could act on spinal neurons through synaptic and network actions, persistent firing and PICs are largely an intrinsic membrane property and the action of motoneurons. This suggests that a two-compartment motoneuron model would be sufficient. The model accounted for the smaller anodal direct effect and the larger cathodal direct and persistent effects we observed experimentally. Further, it also accounted for L-type Ca channel blockade and the loss of the persistent effect, but not the direct effect. The two-compartment model did not capture the very long duration persistent responses after c-tsDCS. Although we were able to model the reduction in the c-tsDCS direct response and elimination of the persistent response to both polarities with a 10% reduction in Ca^2+^ conductance, systematically smaller reductions did not yield progressive response reductions. These findings stress non-linear dynamics underlying tsDCS neuromodulation (Elbasiouny and Mushahwar, 2007). What might contribute to these differences between experimental and modeling effects? The orientation of motoneuron dendritic arbors is heterogeneous. During c-tsDCS, some motoneurons would be expected to experience full cathodal stimulation, whereas others would simultaneously experience anodal stimulation because of differences in their somato-dendritic axes. The particular activity change of each motor unit during tsDCS could be the effect of a combination of c-tsDCS or a-tsDCS depending on its soma-dendritic orientation and susceptibility to polarization by the applied tsDCS. Although most dendrites of motoneurons are located dorsal to the soma (Balaskas et al., 2019), with a net orientation parallel to the applied tsDCS field, the soma-dendritic orientation relative to the external field varies (see Figure 6). A multicompartment motoneuron model showed that the non-linear properties of the voltage-gated Ca^2+^ channel could lead to suppression of PICs in both depolarized or hyperpolarized dendritic regions (Elbasiouny and Mushahwar, 2007). However, motoneuron dendrites were modeled with a radially symmetrical morphology around the soma, which may have led to different predictions and did not accord with our experimental results. Considering the single dendritic compartment in the model we developed, it suggests that two dominant factors leading to persistent firing after c-tsDCS are polarization of the dorsally- (or dorsomedially-) directed dendritic arbor and dendritic localization of the Ca channels.

### Clinical Significance of Trans-Spinal Direct Current Stimulation for Rehabilitation After Injury

Phasic activation of spinal motor circuits holds much promise to improve motor function in humans after spinal cord injury (SCI) (Angeli et al., 2014, 2018; Pena Pino et al., 2020), and the effects of a similar phasic stimulation showed a frequency and segmental-level dependence (Vogelstein et al., 2006). Phasic spinal stimulation is thought to activate CPGs, possibly through activation of large-diameter proprioceptive inputs (Formento et al., 2018). Moreover, non-invasive phasic stimulation methods are being developed to target the human spinal cord after injury (Inanici et al., 2021). The noninvasive application of tsDCS using surface electrodes is appropriate for behaving animals and humans. By developing an understanding of the molecular mechanisms of tsDCS, we can better inform therapeutic strategies of this method for promoting function after spinal injury. The specific effect of augmenting PIC-like responses with c-tsDCS is a novel molecular target. It must be stressed that PIC-like responses are produced during naturally-occurring motor actions in rats and humans (Kiehn and Eken, 1989; Gorassini et al., 2002a,b). PICs are regarded to be an important component of normal motor control and, together with a wide-range of channel types in motoneurons, offer extraordinary flexibility for muscle force control (Heckmann et al., 2005). Enhancing the PIC-like response of motor units induced by c-tsDCS would be well-suited to potentiate motor strength after SCI.

After complete sacral transection, the 5-HT2_C_ receptor caudal to injury can become constitutively active, resulting in unregulated PICs that contribute to hyperreflexia and spasms (Murray et al., 2010). This is thought to reflect the loss of descending monoaminergic regulation of motoneuronal excitability. Since hyperreflexia and spasticity are considered to reflect enhances spinal excitability, would c-tsDCS exacerbate these conditions in the injured spinal cord? Or through its targeted use to counter the loss of descending excitatory control signals, might it interrupt the circle of loss of excitability leading to maladaptive hyperreflexia that occurs after SCI, and especially after perinatal corticospinal system injury (Cavarsan et al., 2019; Steele et al., 2020)? The mechanism for PIC-like motor unit firing induced by c-tsDCS, L-type Ca^2+^ channel activation, has recently been targeted pharmacologically with Nimodipine to ameliorate spasticity in mice after complete sacral SCI (Marcantoni et al., 2020). This adds to the well-known amelioration of spasticity after rehabilitation (Cote et al., 2014; Beverungen et al., 2020). C-tsDCS neuromodulation enhances PICs, and presumably force capacity, which is necessary to improve motor capacity post-injury. By contrast, Nimodipine reduces PICs and presumably force capacity, thereby exacerbating weakness but ameliorating hyperreflexia. Intriguingly, these two interventions might be recruited in different combinations and different times after motor system injury—as spinal excitability changes evolve and hyperreflexia and spasms develop—to modify PIC production bidirectionally for different rehabilitation goals.

## Data Availability Statement

The original contributions presented in the study are included in the article/supplementary material, further inquiries can be directed to the corresponding author/s.

## Ethics Statement

The animal study was reviewed and approved by City University of New York Advanced Science Research Center IACUC.

## Author Contributions

WS performed the research and analyzed the data. JM supervised all aspects of work. Both authors designed the research, wrote the manuscript, and approved the submitted version.

## Conflict of Interest

The authors declare that the research was conducted in the absence of any commercial or financial relationships that could be construed as a potential conflict of interest.

## Publisher’s Note

All claims expressed in this article are solely those of the authors and do not necessarily represent those of their affiliated organizations, or those of the publisher, the editors and the reviewers. Any product that may be evaluated in this article, or claim that may be made by its manufacturer, is not guaranteed or endorsed by the publisher.

## References

[B1] Abualait T. S. (2020). Effects of transcranial direct current stimulation of primary motor cortex on cortical sensory deficits and hand dexterity in a patient with stroke: a case study. *J. Int. Med. Res.* 48:300060519894137. 10.1177/0300060519894137 31885346 PMC7783281

[B2] Aguilar J. Pulecchi F. Dilena R. Oliviero A. Priori A. Foffani G. (2011). Spinal direct current stimulation modulates the activity of gracile nucleus and primary somatosensory cortex in anaesthetized rats. *J. Physiol.* 589 4981–4996. 10.1113/jphysiol.2011.214189 21825031 PMC3224887

[B3] Ahmed Z. (2011). Trans-spinal direct current stimulation modulates motor cortex-induced muscle contraction in mice. *J. Appl. Physiol.* 110 1414–1424. 10.1152/japplphysiol.01390.2010 21350028

[B4] Ahmed Z. (2013a). Effects of cathodal trans-spinal direct current stimulation on mouse spinal network and complex multijoint movements. *J. Neurosci.* 33 14949–14957. 10.1523/JNEUROSCI.2793-13.2013 24027294 PMC6705168

[B5] Ahmed Z. (2013b). Electrophysiological characterization of spino-sciatic and cortico-sciatic associative plasticity: modulation by trans-spinal direct current and effects on recovery after spinal cord injury in mice. *J. Neurosci.* 33 4935–4946. 10.1523/JNEUROSCI.4930-12.2013 23486964 PMC6619000

[B6] Ahmed Z. (2014). Trans-spinal direct current stimulation alters muscle tone in mice with and without spinal cord injury with spasticity. *J. Neurosci.* 34 1701–1709. 10.1523/JNEUROSCI.4445-13.2014 24478352 PMC6827582

[B7] Ahmed Z. (2016). Modulation of gamma and alpha spinal motor neurons activity by trans-spinal direct current stimulation: effects on reflexive actions and locomotor activity. *Physiol. Rep.* 4:e12696. 10.14814/phy2.12696 26869682 PMC4758926

[B8] Angeli C. A. Boakye M. Morton R. A. Vogt J. Benton K. Chen Y. (2018). Recovery of over-ground walking after chronic motor complete spinal cord injury. *N. Engl. J. Med.* 379 1244–1250. 10.1056/NEJMoa1803588 30247091

[B9] Angeli C. A. Edgerton V. R. Gerasimenko Y. P. Harkema S. J. (2014). Altering spinal cord excitability enables voluntary movements after chronic complete paralysis in humans. *Brain* 137 1394–1409. 10.1093/brain/awu038 24713270 PMC3999714

[B10] Baczyk M. Drzymala-Celichowska H. Mrowczynski W. Krutki P. (2019). Motoneuron firing properties are modified by trans-spinal direct current stimulation in rats. *J. Appl. Physiol. (1985)* 126 1232–1241. 10.1152/japplphysiol.00803.2018 30789288

[B11] Baczyk M. Drzymala-Celichowska H. Mrowczynski W. Krutki P. (2020b). Polarity-dependent adaptations of motoneuron electrophysiological properties after 5-wk transcutaneous spinal direct current stimulation in rats. *J. Appl. Physiol. (1985)* 129 646–655. 10.1152/japplphysiol.00301.2020 32790599

[B12] Baczyk M. Drzymala-Celichowska H. Mrowczynski W. Krutki P. (2020a). Long-lasting modifications of motoneuron firing properties by trans-spinal direct current stimulation in rats. *Eur. J. Neurosci.* 51 1743–1755. 10.1111/ejn.14612 31677210

[B13] Baczyk M. Pettersson L. G. Jankowska E. (2014). Facilitation of ipsilateral actions of corticospinal tract neurons on feline motoneurons by transcranial direct current stimulation. *Eur. J. Neurosci.* 40 2628–2640. 10.1111/ejn.12623 24835584 PMC4142254

[B14] Balaskas N. Abbott L. F. Jessell T. M. Ng D. (2019). Positional strategies for connection specificity and synaptic organization in spinal sensory-motor circuits. *Neuron* 102 1143–1156 e1144. 10.1016/j.neuron.2019.04.008 31076274 PMC7085297

[B15] Beverungen H. Klaszky S. C. Klaszky M. Cote M. P. (2020). Rehabilitation decreases spasticity by restoring chloride homeostasis through the brain-derived neurotrophic factor-KCC2 pathway after spinal cord injury. *J. Neurotrauma* 37 846–859. 10.1089/neu.2019.6526 31578924 PMC7071070

[B16] Bikson M. Inoue M. Akiyama H. Deans J. K. Fox J. E. Miyakawa H. (2004). Effects of uniform extracellular DC electric fields on excitability in rat hippocampal slices in vitro. *J. Physiol.* 557 175–190. 10.1113/jphysiol.2003.055772 14978199 PMC1665051

[B17] Bocci T. Marceglia S. Vergari M. Cognetto V. Cogiamanian F. Sartucci F. (2015). Transcutaneous spinal direct current stimulation modulates human corticospinal system excitability. *J. Neurophysiol.* 114 440–446. 10.1152/jn.00490.2014 25925328 PMC4509392

[B18] Bocci T. Vannini B. Torzini A. Mazzatenta A. Vergari M. Cogiamanian F. (2014). Cathodal transcutaneous spinal direct current stimulation (tsDCS) improves motor unit recruitment in healthy subjects. *Neurosci. Lett.* 578 75–79. 10.1016/j.neulet.2014.06.037 24970753

[B19] Bolzoni F. Jankowska E. (2015). Presynaptic and postsynaptic effects of local cathodal DC polarization within the spinal cord in anaesthetized animal preparations. *J. Physiol.* 593 947–966. 10.1113/jphysiol.2014.285940 25416625 PMC4398531

[B20] Bolzoni F. Baczyk M. Jankowska E. (2013). Subcortical effects of transcranial direct current stimulation in the rat. *J. Physiol.* 591 4027–4042. 10.1113/jphysiol.2013.257063 23774279 PMC3764643

[B21] Booth V. Rinzel J. Kiehn O. (1997). Compartmental model of vertebrate motoneurons for Ca2+-dependent spiking and plateau potentials under pharmacological treatment. *J. Neurophysiol.* 78 3371–3385. 10.1152/jn.1997.78.6.3371 9405551

[B22] Button D. C. Gardiner K. Marqueste T. Gardiner P. F. (2006). Frequency-current relationships of rat hindlimb alpha-motoneurones. *J. Physiol.* 573 663–677. 10.1113/jphysiol.2006.107292 16613880 PMC1779753

[B23] Carlson A. P. Hanggi D. Macdonald R. L. Shuttleworth C. W. (2020). Nimodipine reappraised: an old drug with a future. *Curr. Neuropharmacol.* 18 65–82. 10.2174/1570159X17666190927113021 31560289 PMC7327937

[B24] Cavarsan C. F. Gorassini M. A. Quinlan K. A. (2019). Animal models of developmental motor disorders: parallels to human motor dysfunction in cerebral palsy. *J. Neurophysiol.* 122 1238–1253. 10.1152/jn.00233.2019 31411933 PMC6766736

[B25] Cote M. P. Gandhi S. Zambrotta M. Houle J. D. (2014). Exercise modulates chloride homeostasis after spinal cord injury. *J. Neurosci.* 34 8976–8987. 10.1523/JNEUROSCI.0678-14.2014 24990918 PMC6608257

[B26] Elbasiouny S. M. Mushahwar V. K. (2007). Suppressing the excitability of spinal motoneurons by extracellularly applied electrical fields: insights from computer simulations. *J. Appl. Physiol. (1985)* 103 1824–1836. 10.1152/japplphysiol.00362.2007 17702836

[B27] Formento E. Minassian K. Wagner F. Mignardot J. B. Le Goff-Mignardot C. G. Rowald A. (2018). Electrical spinal cord stimulation must preserve proprioception to enable locomotion in humans with spinal cord injury. *Nat. Neurosci.* 21 1728–1741. 10.1038/s41593-018-0262-6 30382196 PMC6268129

[B28] Fritsch B. Reis J. Martinowich K. Schambra H. M. Ji Y. Cohen L. G. (2010). Direct current stimulation promotes BDNF-dependent synaptic plasticity: potential implications for motor learning. *Neuron* 66 198–204. 10.1016/j.neuron.2010.03.035 20434997 PMC2864780

[B29] Gorassini M. Bennett D. J. Kiehn O. Eken T. Hultborn H. (1999). Activation patterns of hindlimb motor units in the awake rat and their relation to motoneuron intrinsic properties. *J. Neurophysiol.* 82 709–717. 10.1152/jn.1999.82.2.709 10444668

[B30] Gorassini M. Yang J. F. Siu M. Bennett D. J. (2002a). Intrinsic activation of human motoneurons: possible contribution to motor unit excitation. *J. Neurophysiol.* 87 1850–1858. 10.1152/jn.00024.2001 11929906

[B31] Gorassini M. Yang J. F. Siu M. Bennett D. J. (2002b). Intrinsic activation of human motoneurons: reduction of motor unit recruitment thresholds by repeated contractions. *J. Neurophysiol.* 87 1859–1866. 10.1152/jn.00025.2001 11929907

[B32] Guidetti M. Ferrucci R. Vergari M. Aglieco G. Naci A. Versace S. (2021). Effects of transcutaneous spinal direct current stimulation (tsDCS) in patients with chronic pain: a clinical and neurophysiological study. *Front. Neurol.* 12:695910. 10.3389/fneur.2021.695910 34552550 PMC8450534

[B33] Heckman C. J. Lee R. H. Brownstone R. M. (2003). Hyperexcitable dendrites in motoneurons and their neuromodulatory control during motor behavior. *Trends Neurosci.* 26 688–695. 10.1016/j.tins.2003.10.002 14624854

[B34] Heckmann C. J. Gorassini M. A. Bennett D. J. (2005). Persistent inward currents in motoneuron dendrites: implications for motor output. *Muscle Nerve* 31 135–156. 10.1002/mus.20261 15736297

[B35] Hernandez-Labrado G. R. Polo J. L. Lopez-Dolado E. Collazos-Castro J. E. (2011). Spinal cord direct current stimulation: finite element analysis of the electric field and current density. *Med. Biol. Eng. Comput.* 49 417–429. 10.1007/s11517-011-0756-9 21409426

[B36] Hultborn H. (2002). Plateau potentials and their role in regulating motoneuronal firing. *Adv. Exp. Med. Biol.* 508 213–218. 10.1007/978-1-4615-0713-0_26 12171114

[B37] Ievins A. Moritz C. T. (2017). Therapeutic stimulation for restoration of function after spinal cord injury. *Physiology (Bethesda)* 32 391–398. 10.1152/physiol.00010.2017 28814499

[B38] Inanici F. Brighton L. N. Samejima S. Hofstetter C. P. Moritz C. T. (2021). Transcutaneous spinal cord stimulation restores hand and arm function after spinal cord injury. *IEEE Trans. Neural Syst. Rehabil. Eng.* 29 310–319. 10.1109/TNSRE.2021.3049133 33400652

[B39] Jack A. S. Hurd C. Martin J. Fouad K. (2020). Electrical stimulation as a tool to promote plasticity of the injured spinal cord. *J. Neurotrauma* 37 1933–1953. 10.1089/neu.2020.7033 32438858 PMC7470222

[B40] Jankowska E. (2017). Spinal control of motor outputs by intrinsic and externally induced electric field potentials. *J. Neurophysiol* 118 1221–1234. 10.1152/jn.00169.2017 28539396 PMC5547263

[B41] Jankowska E. Kaczmarek D. Bolzoni F. Hammar I. (2017). Long-lasting increase in axonal excitability after epidurally applied DC. *J. Neurophysiol.* 118 1210–1220. 10.1152/jn.00148.2017 28515284 PMC5547254

[B42] Kiehn O. Eken T. (1989). Bistable firing properties of soleus motor units in unrestrained rats. *Acta Physiol. Scand.* 136 383–394. 10.1111/j.1748-1716.1989.tb08679.x 2750539

[B43] Kim H. (2017). Impact of the localization of dendritic calcium persistent inward current on the input-output properties of spinal motoneuron pool: a computational study. *J. Appl. Physiol. (1985)* 123 1166–1187. 10.1152/japplphysiol.00034.2017 28684585

[B44] Knikou M. Dixon L. Santora D. Ibrahim M. M. (2015). Transspinal constant-current long-lasting stimulation: a new method to induce cortical and corticospinal plasticity. *J. Neurophysiol.* 114 1486–1499. 10.1152/jn.00449.2015 26108955 PMC4556848

[B45] Kurian M. Crook S. M. Jung R. (2011). Motoneuron model of self-sustained firing after spinal cord injury. *J. Comput. Neurosci.* 31 625–645. 10.1007/s10827-011-0324-1 21526348 PMC5036975

[B46] Lafon B. Rahman A. Bikson M. Parra L. C. (2017). Direct current stimulation alters neuronal input/output function. *Brain Stimul.* 10 36–45. 10.1016/j.brs.2016.08.014 27717601 PMC5774009

[B47] Lamy J. C. Varriale P. Apartis E. Mehdi S. Blancher-Meinadier A. Kosutzka Z. (2021). Trans-spinal direct current stimulation for managing primary orthostatic tremor. *Mov. Disord.* 36 1835–1842. 10.1002/mds.28581 33772851

[B48] Lefaucheur J. P. Antal A. Ayache S. S. Benninger D. H. Brunelin J. Cogiamanian F. (2017). Evidence-based guidelines on the therapeutic use of transcranial direct current stimulation (tDCS). *Clin. Neurophysiol.* 128 56–92. 10.1016/j.clinph.2016.10.087 27866120

[B49] Li Y. Bennett D. J. (2003). Persistent sodium and calcium currents cause plateau potentials in motoneurons of chronic spinal rats. *J. Neurophysiol.* 90 857–869. 10.1152/jn.00236.2003 12724367

[B50] Liebetanz D. Koch R. Mayenfels S. Konig F. Paulus W. Nitsche M. A. (2009). Safety limits of cathodal transcranial direct current stimulation in rats. *Clin. Neurophysiol.* 120 1161–1167. 10.1016/j.clinph.2009.01.022 19403329

[B51] Marcantoni M. Fuchs A. Low P. Bartsch D. Kiehn O. Bellardita C. (2020). Early delivery and prolonged treatment with nimodipine prevents the development of spasticity after spinal cord injury in mice. *Sci. Transl. Med.* 12:eaay0167. 10.1126/scitranslmed.aay0167 32295897

[B52] McCreery D. B. Agnew W. F. Yuen T. G. Bullara L. (1990). Charge density and charge per phase as cofactors in neural injury induced by electrical stimulation. *IEEE Trans. Biomed. Eng.* 37 996–1001. 10.1109/10.102812 2249872

[B53] Mekhael W. Begum S. Samaddar S. Hassan M. Toruno P. Ahmed M. (2019). Repeated anodal trans-spinal direct current stimulation results in long-term reduction of spasticity in mice with spinal cord injury. *J. Physiol.* 597 2201–2223. 10.1113/JP276952 30689208 PMC6462463

[B54] Monte-Silva K. Kuo M. F. Hessenthaler S. Fresnoza S. Liebetanz D. Paulus W. (2013). Induction of late LTP-like plasticity in the human motor cortex by repeated non-invasive brain stimulation. *Brain Stimul.* 6 424–432. 10.1016/j.brs.2012.04.011 22695026

[B55] Murray K. C. Nakae A. Stephens M. J. Rank M. D’amico J. Harvey P. J. (2010). Recovery of motoneuron and locomotor function after spinal cord injury depends on constitutive activity in 5-HT2C receptors. *Nat. Med.* 16 694–700. 10.1038/nm.2160 20512126 PMC3107820

[B56] Pena Pino I. Hoover C. Venkatesh S. Ahmadi A. Sturtevant D. Patrick N. (2020). Long-term spinal cord stimulation after chronic complete spinal cord injury enables volitional movement in the absence of stimulation. *Front. Syst. Neurosci.* 14:35. 10.3389/fnsys.2020.00035 32714156 PMC7340010

[B57] Picelli A. Chemello E. Castellazzi P. Roncari L. Waldner A. Saltuari L. (2015). Combined effects of transcranial direct current stimulation (tDCS) and transcutaneous spinal direct current stimulation (tsDCS) on robot-assisted gait training in patients with chronic stroke: a pilot, double blind, randomized controlled trial. *Restor. Neurol. Neurosci.* 33 357–368. 26410579 10.3233/RNN-140474

[B58] Rahman A. Reato D. Arlotti M. Gasca F. Datta A. Parra L. C. (2013). Cellular effects of acute direct current stimulation: somatic and synaptic terminal effects. *J. Physiol.* 591 2563–2578. 10.1113/jphysiol.2012.247171 23478132 PMC3678043

[B59] Song W. Amer A. Ryan D. Martin J. H. (2016). Combined motor cortex and spinal cord neuromodulation promotes corticospinal system functional and structural plasticity and motor function after injury. *Exp. Neurol.* 277 46–57. 10.1016/j.expneurol.2015.12.008 26708732 PMC4807330

[B60] Song W. Martin J. H. (2017). Spinal cord direct current stimulation differentially modulates neuronal activity in the dorsal and ventral spinal cord. *J. Neurophysiol.* 117 1143–1155. 10.1152/jn.00584.2016 28031400 PMC5340879

[B61] Song W. Truong D. Q. Bikson M. Martin J. H. (2015). Transspinal direct current stimulation immediately modifies motor cortex sensorimotor maps. *J. Neurophysiol.* 113 2801–2811. 10.1152/jn.00784.2014 25673738 PMC4416633

[B62] Steele P. R. Cavarsan C. F. Dowaliby L. Westefeld M. Katenka N. Drobyshevsky A. (2020). Altered motoneuron properties contribute to motor deficits in a rabbit hypoxia-ischemia model of cerebral palsy. *Front. Cell Neurosci.* 14:69. 10.3389/fncel.2020.00069 32269513 PMC7109297

[B63] Stifani N. (2014). Motor neurons and the generation of spinal motor neuron diversity. *Front. Cell Neurosci.* 8:293. 10.3389/fncel.2014.00293 25346659 PMC4191298

[B64] Vogelstein R. J. Etienne-Cummings R. Thakor N. V. Cohen A. H. (2006). Phase-dependent effects of spinal cord stimulation on locomotor activity. *IEEE Trans. Neural Syst. Rehabil. Eng.* 14 257–265. 10.1109/TNSRE.2006.881586 17009484

[B65] Winkler T. Hering P. Straube A. (2010). Spinal DC stimulation in humans modulates post-activation depression of the H-reflex depending on current polarity. *Clin. Neurophysiol.* 121 957–961. 10.1016/j.clinph.2010.01.014 20153248

[B66] Yuen T. G. Agnew W. F. Bullara L. A. Jacques S. Mccreery D. B. (1981). Histological evaluation of neural damage from electrical stimulation: considerations for the selection of parameters for clinical application. *Neurosurgery* 9 292–299. 10.1227/00006123-198109000-00013 7301072

